# Inactivation of Infectious Bacteria Using Nonthermal Biocompatible Plasma Cabinet Sterilizer

**DOI:** 10.3390/ijms21218321

**Published:** 2020-11-06

**Authors:** Mahmuda Akter, Dharmendra Kumar Yadav, Se Hoon Ki, Eun Ha Choi, Ihn Han

**Affiliations:** 1 Department of Plasma Bio-Display, Kwangwoon University, Seoul 01897, Korea; nipa21stfeb@gmail.com; 2 Plasma Bioscience Research Center, Applied Plasma Medicine Center, Kwangwoon University, Seoul 01897, Korea; ksh8721@naver.com; 3 Department of Pharmacy, College of Pharmacy, Gachon University of Medicine and Science, Incheon City 21924, Korea; dharmendra30oct@gmail.com; 4 Department of Electronic and Biological Physics, Kwangwoon University, Seoul 01897, Korea

**Keywords:** Nonthermal biocompatible plasma (NBP), cabinet sterilizer, *Escherichia coli (E. coli)*, *Staphylococcus aureus (S. aureus)*, *Salmonella typhimurium* (sepsis), Reactive oxygen species (ROS), Reactive nitrogen species (RNS)

## Abstract

Nonthermal, biocompatible plasma (NBP) is a promising unique state of matter that is effective against a wide range of pathogenic microorganisms. This study focused on a sterilization method for bacteria that used the dielectric barrier discharge (DBD) biocompatible plasma cabinet sterilizer as an ozone generator. Reactive oxygen species play a key role in inactivation when air or other oxygen-containing gases are used. Compared with the untreated control, *Escherichia coli* *(E. coli)*, *Staphylococcus aureus (S. aureus)*, and *Salmonella typhimurium* (sepsis) were inhibited by approximately 99%, or were nondetectable following plasma treatment. Two kinds of plasma sterilizers containing six- or three-chamber cabinets were evaluated. There was no noticeable difference between the two configurations in the inactivation of microorganisms. Both cabinet configurations were shown to be able to reduce microbes dramatically, i.e., to the nondetectable range. Therefore, our data indicate that the biocompatible plasma cabinet sterilizer may prove to be an appropriate alternative sterilization procedure.

## 1. Introduction

The use of physical means, such as atmospheric pressure plasmas, to sterilize surfaces and environments containing pathogenic microorganisms has received considerable attention in the past decades. Sterilization is defined as the total inactivation of all forms of living microorganisms and their spores. There are several drawbacks to sterilizing therapeutic devices in hospitals using conventional methods, such as wet/dry heat, chemical gases, or irradiation; for example, these approaches may affect the physical and biological performance of the therapeutic device [1], leading to material failure [2]. For successful disinfection, new approaches are required to eliminate infectious diseases. Nonthermal atmospheric pressure plasma technology is a promising potential sterilization method for the treatment of infectious diseases; notably, it does not suffer from the disadvantages of conventional methods [3,4].

Plasma technologies can serve as surface remedies and environmental sterilization approaches. ROS and RNS are produced by plasma, and can combine and destroy the cell surface of bacteria. Several studies have stated that reactive oxygen species play a main function in the inactivation of microbes, while other components generated by plasma (e.g., UV photons, electric fields, charged particles, and heat) make a small contribution [5,6]. NBP has been implemented for cancer treatments [7,8], sterilized viruses, bacteria, and mold [7,9], wound-healing therapies [10,11], etc. Recently, these approaches have been studied for food sterilization and plasma medicine [12,13,14]. Plasma also inactivates an extensive range of microbes through cellular-deadly reactive species [15,16,17]. Furthermore, the inactivation capability may be changed using different working gases, from which completely different types or amounts of reactive species could be generated [18,19,20]. Plasma-generated and the actuated arrangement of localized ROS within the gas and fluid stages consisting of atomic oxygen (O), singlet oxygen (O_2_), ozone (O_3_), hydroxyl radicals (**•**OH), hydrogen peroxide (H_2_O_2_) may destroy the bacterial structure through different pathways [21,22,23,24]. Inactivation was accomplished with diverse plasma sources such as a plasma jet, dielectric barrier discharge (DBD), microwave discharge, corona discharges, and plasma coverage [4]. Ehlbeck et al. studied the effect of plasma on the growth of approximately 20 forms of gram-positive and -negative pathogenic microbes. Other studies on microbial sterilization using nonthermal atmospheric plasma have been conducted worldwide [12,25,26,27,28]. Several research groups have established the inactivation of endospores [29,30], and many groups have presented effective microbial sterilization by atmospheric pressure plasma [31,32].

In this study, we report on the sterilization effect of a plasma cabinet sterilizer on *Escherichia coli, staphylococcus aureus,* and *Salmonella typhimurium*. These strains were selected as they are well-characterized gram-negative and -positive microbes that are utilized as model bacteria owing to their speedy growth rates. There are many reviews on the use of plasmas in sterilization applications; such a body of research is usually a prerequisite for the application of a sterilization procedure, as defined in global standards [32].

## 2. Results

### 2.1. Physical Characterization and RONS Generation of DBD Plasma Cabinet Sterilizer

**Figure 1:**
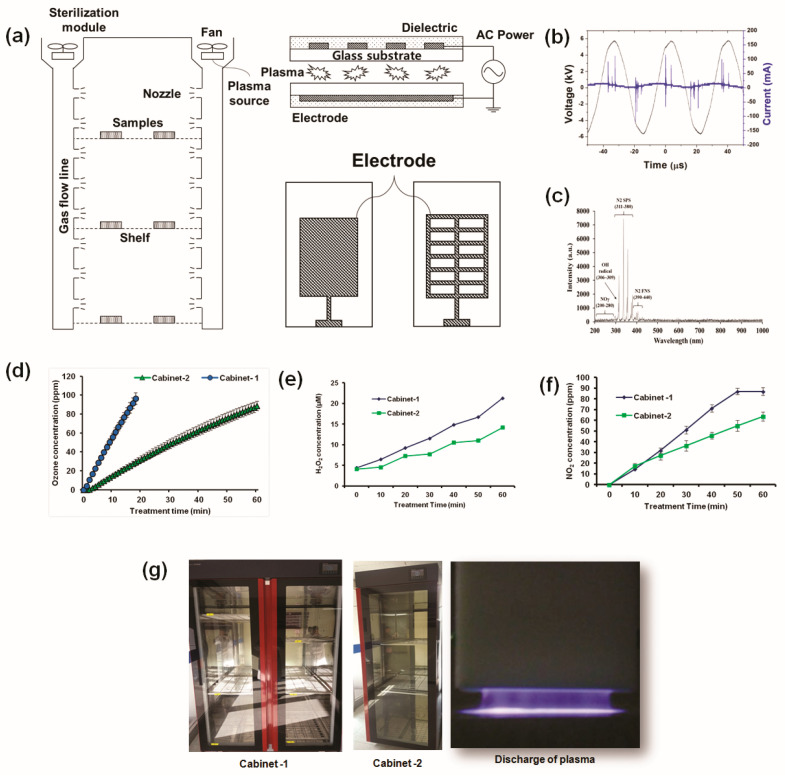
(**a**) Schematic of the experimental setup with the DBD nonthermal biocompatible plasma electrode structure; (**b**) current and voltage waveforms during discharge; (**c**) optical emission spectra (OES) of plasma; (**d**) ozone concentration measurement during treatment time; (**e**) the amount of H_2_O_2_; and (**f**) NO_2_ concentration according to plasma treatment time. (**g**) Photograph of the plasma cabinet sterilizer with six and three chambers and the discharge photo of plasma.

Figure 1a shows a schematic configuration of the three-chamber cabinet system at atmospheric pressure under an air gas flow. The nonthermal plasma cabinet used in this experiment was made by Plasma Bioscience Research Center and Dawoo Korea (details in Materials and Methods Section). Typical waveforms of the current and voltage discharge created in the DBD plasma cabinet are shown in Figure 1b. As shown in Table 1, the electric discharge current was ∼13.01 mA for Irms at a frequency of 27.6 kHz with a Vrms voltage of 4.16 kV.

To measure the reactive oxygen or nitrogen species formed by the plasma, the optical emission spectrum (OES) was measured between 200 and 1000 nm from the plasma source. The intensity of the light emitted from the device was recorded with respect to the wavelength. The OES of the DBD plasma cabinet device used in this experiment showed that the N_2_ s positive system (SPS) lines were the predominant peaks in the near-UV region (310–380 nm). OH radicals (306–309 nm), N_2_ first negative system (FNS) (390–440 nm), and NOγ (200–280 nm) were also detected weakly in the OES spectrum shown in Figure 1c.

The optical emission spectrum of the DBD plasma emission lines corresponded to N_2_, atomic nitrogen, and hydroxyl radicals. The emission bands produced by the radiative transition of the second positive and first negative systems of the excitation molecular nitrogen were observed between 310 and 440 nm. These OES results explain the ozone generation in Figure 1d; ozone was generated according to the following equation.

(1) $$N_{2}+\;e\;\to \;N_{2}^{+}+\;e\;$$

(2) $$N_{2}^{+}+O_{2}\;\to \;N_{2}+2O\;$$

(3) $$N_{2}^{+}+O_{2}\;\to \;N_{2}O+O$$

(4) $$O+O_{2}+\;M\;\to {\;O}_{3}+M$$

(5) $$\mathrm{OH}\cdot +\;\mathrm{OH}\cdot \;\to {\;H}_{2}O_{2}$$

The ozone produced by the plasma is recognized as a reactive species having a sterilizing effect on microbes [33]. As the amounts of O_3_, H_2_O_2,_ and NO_2_ are known to be modulated by different treatments, we calculated their generated quantities for the different treatment conditions. Figure 1d shows that when plasma was treated for 10 min, the ozone concentration was about 14.8 ppm for the three-chamber (cabinet 2) plasma cabinet, but for 10 min of treatment in the six-chamber (cabinet 1) cabinet, the ozone concentration was 55.7 ppm. For the six-chamber cabinet (cabinet 1) after 20 min of treatment, the ozone concentration increased sharply and exceeded the maximum limit of 100 ppm of the ozone meter. For the three-chamber cabinet (cabinet 2), the ozone concentration was approximately 100 ppm after 60 min of treatment. Figure 1e shows the concentrations of H_2_O_2_ generated according to the plasma processing time. H_2_O_2_ is generated as shown in Equation (5). The H_2_O_2_ concentration continuously increased in both cabinets according to the treatment time. After 60 min of plasma treatment, the H_2_O_2_ concentration was 21.28 μM in cabinet 1 and 14.15 μM in cabinet 2. Figure 1f shows that the NO_2_ concentration also increased in both cabinets according to the treatment conditions. However, both cabinets were shown to be able to significantly change bactericidal activity despite the variations in the levels of ozone, H_2_O_2,_ and NO_2_. Therefore, RONS was shown to play a key role in creating oxidative effects from the plasma. Figure 1g shows the experimental cabinet sterilizer and discharge photo of plasma used in this study. 

### 2.2. Effect of Six Chambers (Cabinet 1) Plasma Cabinet Treatment on Inactivation of Bacteria

**Figure 2:**
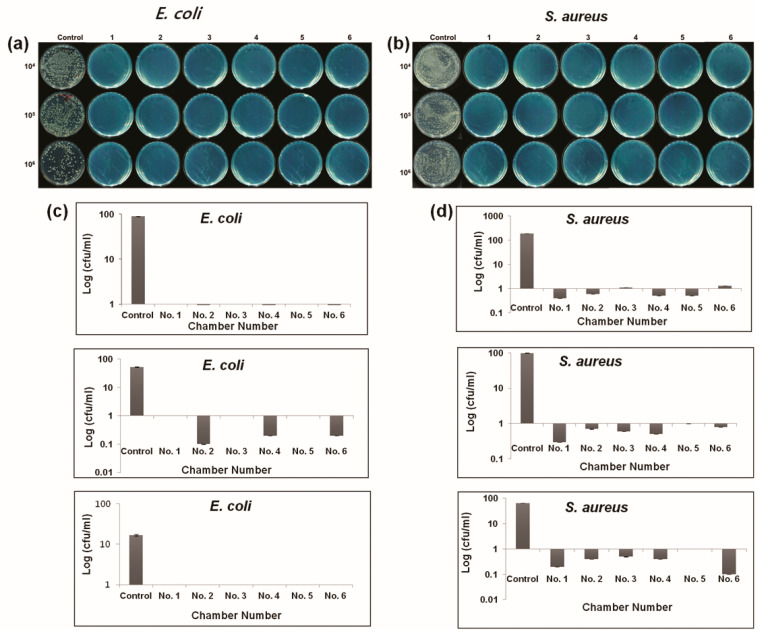
Plasma inactivation efficiency of *Escherichia coli* (*E. coli*) and *Staphylococcus aureus* (*S. aureus*) in the six-chamber (cabinet 1) cabinet (**a**,**b**). Representative culture plates with cultures at 10^4^, 10^5^, and 10^6^ dilution factors. (**c**,**d**) Growth characteristics curve. All *t*-test value is *p* < 0.001 as compared to control.

In this experiment, the goal was to sterilize a therapeutic device. The antibacterial effect of the plasma cabinet sterilizer was evaluated at an optimized temperature, considering the potential effect of low stress and temperature within the cabinet on bacterial viability. To determine the inactivation of *E. coli* and *S. aureus* after plasma sterilization, a conventional cfu counting method involving an agar plate was used (Figure 2) to indicate bacterial culturability. In comparison to the untreated control test, the treated groups exhibited significant deactivation of both bacteria. Specifically, plasma exposure inhibited bacteria growth by almost 99% at concentrations of 1 × 10^6^ and 1 × 10^7^ cfu·ml^−1^; however, for *E coli*, a reduction of 100% was obtained for 1 × 10^8^ cfu·mL^−1^ (Figure 2) compared to the control. The mean log reduction obtained for both species at a 10^4–6^ dilution was plotted to acquire the nondetectable levels of bacteria. For both species, at a 10^6^ dilution, the log reduction was almost zero cfu/mL or nondetectable in the plasma cabinet sterilizer treatment group compared to the control. The error bars for the mean graphs were calculated and plotted based on the standard deviation. Table 2 shows the percentage of inhibition of metabolically active *E. coli* increased from 99.6 ± 0.1% to 99.8 ± 0. 1% and that of *S. aureus* from 98.9 ± 0.1 to 99.8 ± 0.1, or to a nondetectable degree. The rates of inhibition with metabolic capability exhibited upward trends for each microorganism.

### 2.3. Effect of Three-Chamber (cabinet 2) Plasma Cabinet Treatment on the Inactivation of Bacteria

**Figure 3:**
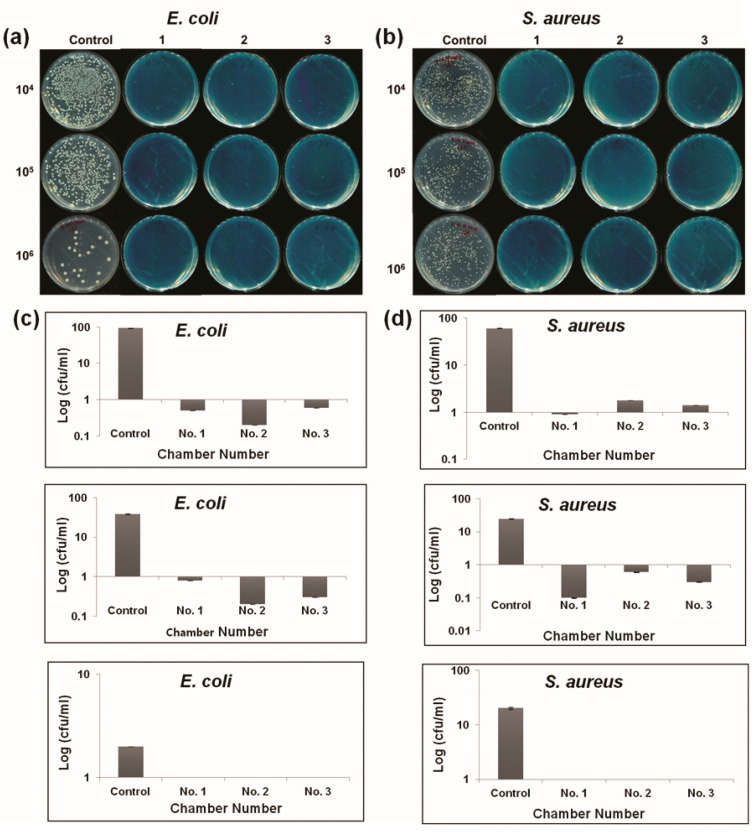
(**a**,**b**) Representative culture plates showing the inactivation efficiency of *Escherichia coli (E. coli)* and *Staphylococcus aureus* (*S. aureus*) at 10^4^, 10^5^, and 10^6^ dilution factors. (**c**,**d**) Growth characteristics curve for the bacteria in the three chambers (cabinet 2) of the plasma cabinet. All *t*-test value is *p* < 0.001 as compared to control.

The efficacy of the three-chamber plasma cabinet in inactivating *E. coli* and *S. aureus* is shown in Figure 3 and (Table 3). For the three-chamber cabinet, the *E. coli* and *S. aureus* bacterial populations were nondetectable in 1 × 10^8^ cfu·mL^−1^ (Table 3). The log reduction was almost zero cfu/mL or nondetectable in the 10^6^ dilutions. The concentrations of each of the bacterial species were observed until they were nondetectable by plate count. It is evident from the table that the population intensity of the bacterial test for the plasma treatment group was reduced for the two microorganisms compared with the control. The difference between the treatment and control groups was significant (*p* < 0.001). The error bars for the mean graphs were measured in the same way as with the six chambers. The inhibition percentage of metabolically active *E. coli* increased from 97.9 ± 0.1% to 99.8 ± 0.1% and *S. aureus* from 97.1 ± 0.1 to 99.6 ± 0.1 or to nondetectable degree, as shown in Table 3. The toxicity of the *Salmonella typhimurium* (sepsis) bacterial inactivation by the cabinet is also shown in the agar plate (Figure S1). As there was no significant difference between the two cabinets, we tested *Salmonella typhimurium* only in the three-chamber cabinet. After plasma treatment, the cfu/mL was less than 10, or was 99.9% inhibited, whereas the control was 1.11 × 10^4^.

### 2.4. In Silico Study

**Figure 4:**
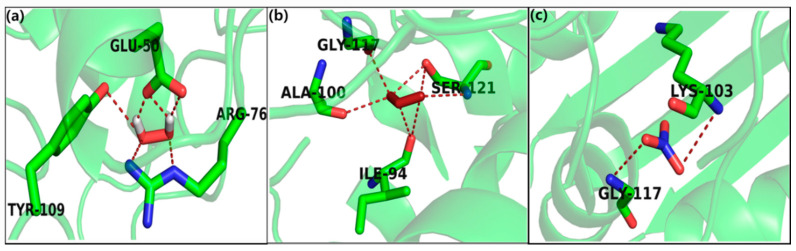
Molecular docking interaction of docked (**a**) hydrogen peroxide, (**b**) ozone, and (**c**) nitrates with *E. coli.*

To assess the stability and reliability underlying the molecular interactions, top docked poses were considered for analysis. The docking results for H_2_O_2_ against *E. coli* (PDB ID: 4PRV) showed a high binding affinity docking score, i.e., −6.512 (Figure 4a), and the formation of six H-bonds to the acidic, (polar, negative charged), e.g., Glu-50 (glutamic acid); positively charged, e.g., Arg4 (arginine); and aromatic (hydrophobic), e.g., Tyr-109 (tyrosine), which are involved in the binding of ROS/RNS and may modulate the function of the protein. Similarly, the docking results of ozone showed high binding affinity, i.e., −6.428 (Figure 4b), and revealed seven H-bonds with hydrophobic amino acid residue, that is, Gly-117 (glycine), Ala-100 (alanine), Ile-94 (isoleucine) and nucleophilic (polar, hydrophobic), that is, SER-10, SER-121 (serine) bound to the protein. Likewise, the nitrates also showed good binding affinity, i.e., −4.562 and three H-bonds with hydrophobic, that is, Gly-117 (glycine) and Lys-103 (lysine); therefore, H_2_O_2_ showed strong hydrophobic interaction with E. coli, which led to more stability and activity in these molecules (Figure 4c).

**Figure 5:**
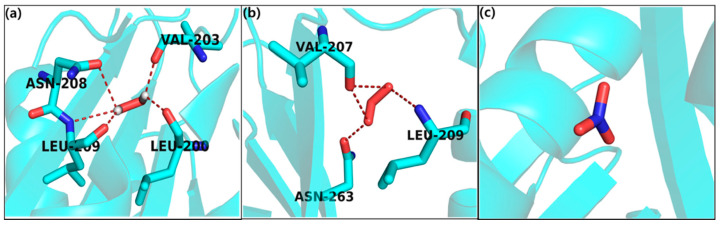
Molecular docking interaction of docked (**a**) hydrogen peroxide, (**b**) ozone, and (**c**) nitrates with *S. aureus*.

On the other hand, the docking results of H_2_O_2_ against the *S. aureus* target protein showed high binding affinity, as indicated by a docking score of −5.7584 (Figure 5a) and the formation of five H-bonds to the hydrophobic, that is, Val-203 (valine); polar amide chemical nature due to Asn-208 (asparagines) and basic (polar, hydrophobic and positive charged), that is, Leu-200, Leu-209 (leucine) within a 3Å radius. Similarly, the docking results for ozone showed high binding affinity, i.e., −5.384 (Figure 5b) and the three H-bonds with hydrophobic polar amide chemical nature due to Asn-263 (asparagines); hydrophobic that is, Val-203 (valine) and basic (polar, hydrophobic and positive charged), that is, Leu-209 bound to the protein. Likewise, the nitrates showed low binding affinity −2.15 without any h-bond interaction (Figure 5c).

**Figure 6:**
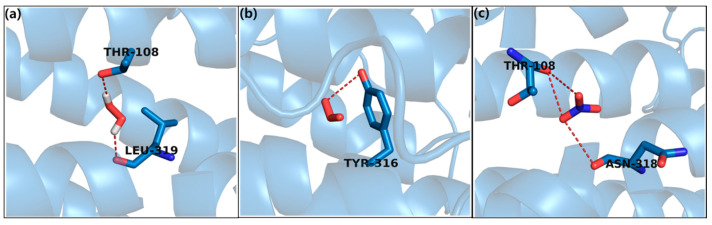
Molecular docking interaction of docked (**a**) hydrogen peroxide, (**b**) ozone, and (**c**) nitrates with *Salmonella typhimurium.*

To further support the above observations, molecular docking studies of hydrogen peroxide, ozone, and nitrates were carried out by exploring the binding site interacting residues. For the docking study, we used a modeled secondary structure, i.e., *S. typhimurium*, which was dominated with an alpha helix (57.94%), followed by random coils (30.29%) and extended strands (8.24%). The details are provided in Supplementary Table S1. Likewise, the docking results for H_2_O_2_ against *S. typhimurium* showed a high binding affinity docking score, i.e., −5.72 (Figure 6a), and the formation of two H-bonds to the nucleophilic (polar, hydrophobic), for example, Thr108 (Threonine) and hydrophobic, for example, Leu-319 (Leucine) were bound to the protein. Model of *S. typhimurium* indicates 82.4% of the residues in the most favourable region, 13.4% in the allowed region, 1.3% in the generously allowed region and 2.9% in the disallowed region (Supplementary Figures S2 and S3).

Similarly, the docking results of ozone showed high binding affinity, i.e., −5.52 (Figure 6b), and a H-bond with nucleophilic (polar, hydrophobic) for example, Thr108. Finally, the nitrates also showed good binding affinity, i.e., −5.94 (Figure 6c), and the formation of three H-bonds with nucleophilic (polar, hydrophobic) for example, Thr108 and polar amide type, for example, Asn318 (Asparagine); were bound to the protein. Therefore, H_2_O_2_, ozone, and nitrates showed strong hydrophobic interactions with *S. typhimurium*, leading to more stability and activity in these molecules.

The simulation study revealed that hydrogen peroxide, ozone, and nitrates showed strong H-bonding and high binding affinity with pathogenic strains *E. coli, S. aureus,* and *Salmonella typhimurium*. Only ozone showed a low binding affinity with *S. aureus*. This fact indicates more potential inhibitory activity against the *E. coli*, *S. aureus and Salmonella typhimurium*. Hence, this approach represents a remarkable milestone in increasing the utility of NBP for plasma medicines.

## 3. Discussion

In this study, sterilization processes for controlling the development of *E. coli, S. aureus*, and *Salmonella typhimurium* (sepsis) were performed. From the inactivation efficacy results, it was shown that plasma has a strong effect on plate count; the inhibition percentage results demonstrated that it reduced bacterial cell counts to nondetectable levels (Figure 2 and Figure 3, and Table 2 and Table 3). This may be due to oxidative pressure due to the reactive species produced by the plasma. Reactive oxygen species can create oxidative stress in cells. In our previous work [34,35], it was reported that ROS and RNS can affect the membranes of cells and microorganisms. During plasma treatment, the generated reactive oxygen species, which are associated with the process and structure parameters, attacked both the cellular envelope and intracellular components.

The atmospheric air plasma creates ROS and RNS species, including hydrogen peroxide, ozone, and nitrates, which are among the foremost commonly identified species utilizing the dielectric barrier discharge (DBD) system [36]. The oxidative damage of macromolecules like DNA, proteins, and lipids by reactive species created by plasma has an inhibitory impact on bacterial populations [37]. Interactions between plasma species and bacteria have already been reported [36].

The majority of plasma sterilizers produce ozone, which can oxidize the cell walls of micro-organisms; then, the plasma cocktail leads to an increase in cell membrane permeability and cell death. This plasma cabinet produces of a variety of different free radicals such as hydroxyl radicals, singlet oxygen, hydrogen peroxide, etc., and not only ozone. The device produces NO and NOx, as well as a small amount of UV. These excited nitrogen molecules can combine with oxygen molecules to form secondary reactive radicals which are effective in damaging the cell membranes. In particular, the device produces hydrogen peroxide gas molecules (H_2_O_2_) which decompose into a plasma state, generating hydroxyl radicals. These are powerful oxidizing agents, which are effective against the cell membranes of microbes. Hydrogen peroxide gas breaks down into water and oxygen after sterilization.

The antimicrobial effects of our nonthermal plasma sterilizer have been successfully demonstrated on different microorganisms. The present study revealed that pathogenic strains can be effectively eradicated using a plasma cabinet. The results obtained for the plasma cabinet sterilizer show that both six- and three-chamber cabinets were effective in inhibiting both pathogenic strains. There was no clear difference in the impact it had on the gram-negative (e.g., *E. coli,* *Salmonella typhimurium* (sepsis) and -positive (e.g., *S. aureus*) bacteria in either cabinet. It was found that the three bacterial strains were sensitive to the plasma cabinet sterilizer based on large and clear growth-inhibition percentages, which suggests that plasma can be used to eradicate a wide range of both gram-positive and -negative bacteria (Table 2 and Table 3). Plasma technology is used in various fields including semiconductors, materials, and agriculture. Plasma-treated carboxymethyl cellulose-coated polypropylene (PP/CMC) films and materials can serve as food antimicrobial packaging [38]. Recent developments, e.g., platforms of nanoparticles, have been used to increase the effect of pharmaceutical formulations for wound healing and functionalized with diverse antimicrobial compounds [39]. Nhan et al. studied the chemical composition of the essential oil extracted from leaves to assess their potential antimicrobial, antitrichomonas and antiviral activities [40]. Overall, the obtained results suggested that the plasma cabinet sterilizer has a high degree of antimicrobial activity against both gram-positive and -negative bacteria. These results also provide fundamental insights into the mechanisms of plasma species interacting with bacteria and into plasma sterilization in general.

## 4. Materials and Methods

### 4.1. Bacterial Strains and Maintenance of Culture

*Escherichia. coli, Staphylococcus aureus,* and *Salmonella typhimurium* strain were used for this study. The strains were selected to represent both gram-negative and -positive bacteria. The strains were obtained from the Korean Collection for Type Cultures (KCTC). The strains were maintained as frozen stocks at −80 °C in an appropriate medium supplemented with 50% glycerol. Each bacterial strain was streaked onto luria bertani agar (LB agar, Agar Bacteriological, MB-A1651, MB cell) and incubated at 37 °C for 24 h. The bacterial culture plates were then maintained at 4 °C.

### 4.2. Preparation of Bacterial Cell Suspension

Cells were grown overnight by inoculating isolated colonies of bacteria in luria bertani medium (LB Broth High Salt, MB-L4488, MB cell) at 37 °C on a rotary shaker at a constant speed of 200 rpm. We estimated cell density using readings of optical density (OD) measurements. Bacterial cells were adjusted to 10^7^ colony forming units per mL (cfu/mL) by optical density measurement at 600 nm (OD600, BioPhotometry, Eppendorf, Hamburg, Germany). A total of 20 μL of the overnight bacterial cultures were placed in a sterile empty petri dish and kept in the clean bench for 15–20 min to dry completely. We divided 20 μL of 10 drops into 2-μL drops for early drying. Then, the bacteria cultures were placed in the plasma cabinet for treatment (50–70 min). These estimates were validated using cfu measurements of the serially diluted cells.

### 4.3. Experimental Cabinet Sterilizer and Plasma Generation in Cabinet

The size of the six- and three-chamber cabinet was 1960 (H) × 1100 (W) × 750 (D) and 1960 (H) × 680 (W) × 750 (D). A dielectric barrier discharge (DBD) plasma system was used. The plasma device was placed in the upper part of the chamber, and the produced RONS passed through the gas flow region into the chamber. As shown in Figure 1a, the structure of the electrode for DBD plasma was composed of a pair of parallel plate electrodes. Six-micrometer thick electrodes were printed on a glass substrate of 0.7 mm using silver attach screen printing. Following drying, SiO_2_ dielectric with 70–100 μm thickness was printed on the substrate and dried. A lattice pattern was used for the top electrode, and flat one for the bottom electrode. The gap distance between the two electrodes was 1.0 mm. The voltages and currents of the electrodes were measured using a GWINSTEK oscilloscope with a 1000 × voltage probe (P6015A, Tektronix) and a current probe (P6021A, Tektronix). Optical emission spectra (OES) were measured using a spectrometer (HR4000, Ocean Optics). The atmospheric air was used to generate plasma. Samples were placed in different chambers and exposed to plasma for 50 min and 20 min, resting to remove the ozone using a charcoal filter. Plasma was exposed for 50 min because the air had to achieve an equilibrium state. After the plasma cabinet treatment, the samples were diluted in 10 mL LB media and subsequently kept in the rotary shaker for 3 h. Finally, the residual live bacteria and spores were counted. Tests were performed in copy and triplicate. The bacterial suspension with no plasma treatment was reserved as a negative control in a rotary shaker for 3 h.

### 4.4. Measurement of Reactive Oxygen and Nitrogen Species (RONS)

We determined the amounts of ozone, hydrogen peroxide, and NO_2_ species in different treatment conditions of the DBD plasma cabinet. An ozone meter (SKT-9300, 0.1 to 100 ppm, resolution 0.1 ppm) was used to measure the concentration of ozone. Hydrogen peroxide was chemically quantified by a commercially available reagent (QuantiChrom TM, Peroxide Assay Kit, DIOX-250, 585 nm). NO_2_ was detected using a NO_2_ detector (GASTiger2000, 0 to 100 ppm, resolution 0.01 ppm).

### 4.5. Colony-Forming unit Analysis

A colony-forming unit (cfu) is used to quantify viable microorganisms; the results are provided as cfu/mL for each plate. Bacterial development was determined by counting colony growth on the agar plates (standard plate count). To examine the bacterial development, the concentrations of the cells (cfu/mL) were determined using the plate counting method [41]. In this technique, appropriate serial dilutions of the microbial cells were utilized to inoculate LB agar plates. The inoculated plates were then incubated at 37 °C for 24 h. By checking the number of colonies created after incubation and multiplying them with the dilution factor, the number of cells in the primary population was determined as cfu/mL. A total of 0.1 mL of the series of dilutions was pipetted onto agar plates utilizing the spread plate technique. After the surfaces of the agar plates were dry, the plates were incubated at 37 °C for 24 h. All the plates that contained 20 to 300 colonies were identified and the colonies on the entire plate were counted per ml.

### 4.6. In Silico Study

To explore the bioactive sites of ROS and RNS species, including hydrogen peroxide, ozone, and nitrates with amino acids of *E. coli* and *S. aureus*, the AutoDock Vina [42] interface was used for molecular docking. The ROS/RNS species (hydrogen peroxide, ozone, and nitrates) were automatically docked into the binding pocket of a target protein using the Lamarckian genetic algorithm, empirically producing a scoring function. The X-ray crystallographic structures of *E. coli* and *S. aureus* (PDB: 4PRV) [43] and (PDB: 3VOB) [44] protein receptor were taken from the protein data bank and further modified for docking calculations. The accuracy of AutoDock in the prediction of ligand confirmation was assured using the redocking procedure explained in [44,45]. One hundred docking poses for each inhibitor were calculated. Kollman united atom partial charges, AutoDock atom types, and polar only hydrogen atoms were taken into account while preparing the protein [46,47].

### 4.7. Statistical Analysis

All tests were repeated a minimum of three times, and the statistical significance of the difference between the mean values was determined through a standard error evaluation. The results are represented as means ± SD. Statistics from the bacterial development studies were compared using the student t-test, and *p* < 0.05 was used as the significance level [48].

## 5. Conclusions

The plasma cabinet sterilizer therapy represents a new antimicrobial technology for the sterilization of pathogenic microorganisms. The air DBD plasma had significant antimicrobial effects on both the gram-negative and -positive microbes in both cabinets. Our data revealed that plasma cabinet treatment is highly effective at inactivating *E. coli.*, *S. aureus,* and *Salmonella typhimurium* (sepsis). RONS played a key role in the presence of oxidative effects from the plasma, leading to the deterioration of bacterial structures and their mediated features. Therefore, DBD plasma might be a powerful device for the eradication of infectious bacteria. Further study will determine the action on molecular and cellular levels regarding the sterilization/disinfection mechanisms of bacteria.

## Figures and Tables

**Table 1:** Physical parameters and conditions of the nonthermal DBD plasma device.

Parameters	Conditions
Voltage (V_rms_, kV)	4.16
Current (I_rms_, mA)	13.01
Period (µs)	36.20
Frequency (kHz)	27.6
Energy (Duty) (J/sec)	0.24

**Table 2:** Inhibition percentage in six-chamber plasma cabinet treatment compared with control. (ND: Nondetected).

Species	Chamber	Inhibition%	Species	Chamber	Inhibition%
*E. coli* (Dilution10^4^)	Control	0	*S. aureus* (Dilution 10^4^)	Control	0
	No. 1	ND		No. 1	99.8 ± 0.1
	No. 2	99.8 ± 0.1		No. 2	99.7 ± 0.1
	No. 3	ND		No. 3	99.4 ± 0.1
	No. 4	99.8 ± 0.1		No. 4	99.7 ± 0.1
	No. 5	ND		No. 5	99.7 ± 0.1
	No. 6	98.9 ± 0.2		No. 6	99.3 ± 0.1
*E. coli* (Dilution10^5^)	Control	0	*S. aureus* (Dilution 10^5^)	Control	0
	No.1	ND		No. 1	99.7 ± 0.1
	No. 2	99.8 ± 0.1		No. 2	99.3 ± 0.1
	No. 3	ND		No. 3	99.4 ± 0.2
	No. 4	99.6 ± 0.1		No. 4	99.5 ± 0.1
	No. 5	ND		No. 5	98.9 ± 0.1
	No. 6	99.6 ± 0.1		No. 6	99.2 ± 0.2
*E. coli* (Dilution 10^6^)	Control	0	*S. aureus* (Dilution 10^6^)	Control	0
	No. 1	ND		No. 1	99.7 ± 0.1
	No. 2	ND		No. 2	99.4 ± 0.1
	No. 3	ND		No. 3	99.2 ± 0.1
	No. 4	ND		No. 4	99.4 ± 0.1
	No. 5	ND		No. 5	ND
	No. 6	ND		No. 6	99.8 ± 0.1

**Table 3:** Percentage of inhibition after three-chamber plasma cabinet treatment compared with control. (ND: Nondetected).

Species	Chamber	Inhibition%	Species	Chamber	Inhibition%
*E. coli* (Dilution10^4^)	Control	0	*S. aureus* (Dilution 10^4^)	Control	0
	No. 1	99.5 ± 0.1		No. 1	98.5 ± 0.1
	No. 2	99.8 ± 0.1		No. 2	97.1 ± 0.1
	No. 3	99.4 ± 0.1		No. 3	97.7 ± 0.1
*E. coli* (Dilution10^5^)	Control	0	*S. aureus* (Dilution 10^5^)	Control	0
	No. 1	97.9 ± 0.1		No. 1	99.6 ± 0.1
	No. 2	99.5 ± 0.1		No. 2	97.6 ± 0.1
	No. 3	99.2 ± 0.1		No. 3	98.8 ±0.1
*E. coli* (Dilution10^6^)	Control	0	*S. aureus* (Dilution 10^6^)	Control	0
	No. 1	ND		No. 1	ND
	No. 2	ND		No. 2	ND
	No. 3	ND		No. 3	ND

## Supplementary Materials

The following are available online at https://www.mdpi.com/1422-0067/21/21/8321/s1. Figure S1: Representative *Salmonella typhimurium* (sepsis) growth characteristics on an agar plate. No viable colonies were observed after the plasma cabinet treatment (cabinet 2). Figure S2: 3D Structure of *S. typhimurium* modelled through I-TASSER server. Helices are represented in red, sheets in gray and loops in green. Figure S3: Ramachandran plot for validation of *S. typhimurium*. Table S1: Details of secondary structure elements of *S. typhimurium*.
