# Supplementary material for: Inactivation of Infectious Bacteria Using Nonthermal Biocompatible Plasma Cabinet Sterilizer
Source: Int J Mol Sci. 2020 Nov 6;21(21):8321. doi: 10.3390/ijms21218321 (PMC7664273; doi:10.3390/ijms21218321)
Supplement: Supplementary file 1 [file ijms-21-08321-s001.pdf]

Inactivation of infectious bacteria using non-thermal biocompatible plasma cabinet sterilizer

Mahmuda Akter[1,2, §], Dharmendra Kumar Yadav[4, §], Se Hoon Ki[2,3], Eun Ha Choi[1,2,3, *] and Ihn Han[1,2,*]

[1]Department of Plasma Bio-Display, Kwangwoon University, Seoul 01897, Korea
[2]Plasma Bioscience Research Center, Applied Plasma Medicine Center, Kwangwoon University, Seoul 01897 Korea
[3]Department of Electronic and Biological Physics, Kwangwoon University, Seoul 01897, Korea
[4]Department of Pharmacy, College of Pharmacy, Gachon University of Medicine and Science, Incheon City, 21924, Korea
*Correspondence: ehchoi@kw.ac.kr(EH.C), hanihn@kw.ac.kr(I H)
§These authors contributed equally

Supplementary data

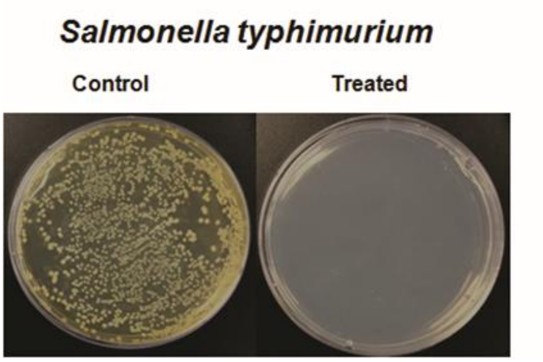

Supplementary Figure S1: Representative *Salmonella typhimurium* (sepsis) growth characteristics on an agar plate. No viable colonies were observed after the plasma cabinet treatment (cabinet 2).

Secondary structures prediction of *Salmonella typhimurium*, using Homology Modelling

Since no crystallographic data is available at present in Protein Data Bank (PDB) (www.rcsb.org) for molecular docking studies. I-TASSER, which is a hierarchical protein structure modelling approach, based on the secondary structure enhanced Profile-Profile threading Alignment (PPA) [1, 2] was used to build the theoretical structure of Salmonella typhi. The sequence of Salmonella typhi containing 340 amino acid residues with accession no Q56136 was retrieved from the universal protein resource (UniProtKB [3]) in FASTA format. The best model was selected based on the C-score (confident score) and validated by PROCHECK [4] from SAVES server.

Homology Modelling and structure validation

From the BLAST results, PDB (Protein Data Bank) structures 3NZZ and 2YM9 showed 85% and 87% identity identity respectively with *S. typhimurium*, and were used as templates for modelling through I-TASSER server where the modelled *S. typhimurium* possessed a C-Score of −0.65 (Supplementary Fig. 1). C-score is a confidence score for estimating the quality of predicted models by I-TASSER. It was calculated based on the significance of threading template alignments and the convergence parameters of the structure assembly simulations. Ramachandran plot calculations were estimated through PROCHECK program. Model of *S. typhimurium* indicates 82.4% of the residues in the most favourable region, 13.4% in the allowed region, 1.3% in the generously allowed region and 2.9% in the disallowed region (Supplementary Fig. S2).

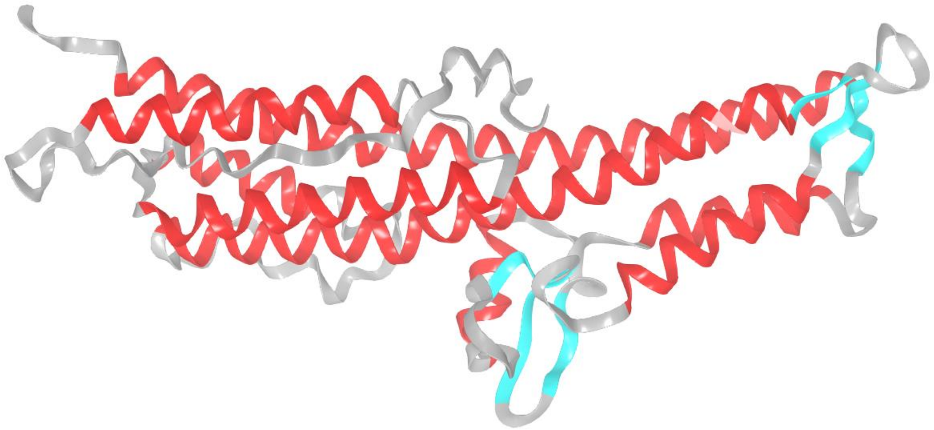

Supplementary Figure S2: 3D Structure of *S. typhimurium* modelled through I-TASSER server. Helices are represented in red, sheets in gray and loops in green

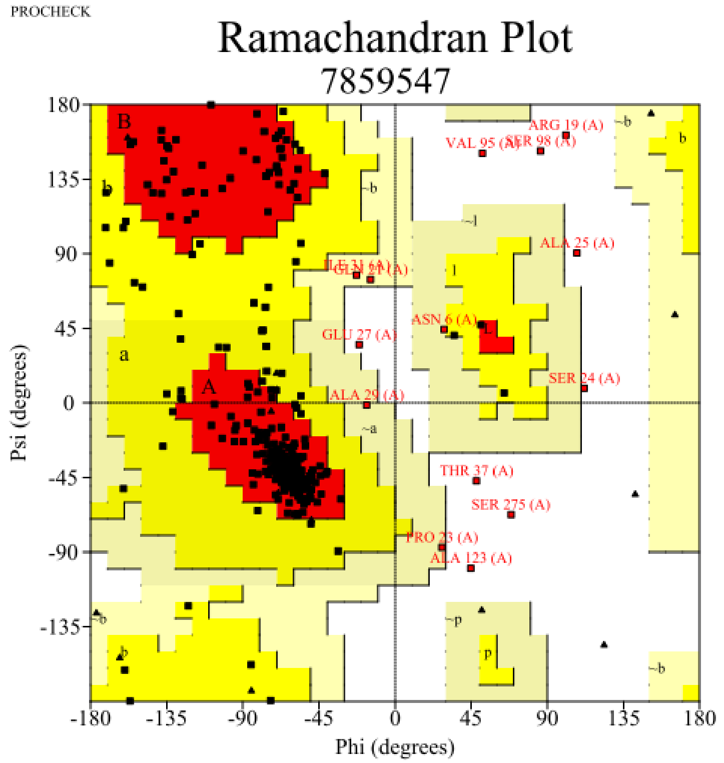

Plot statistics

Residues in most favoured regions [A,B,L]	252	82.4%
Residues in additional allowed regions [a,b,l,p]	41	13.4%
Residues in generously allowed regions [~a,~b,~l,~p]	4	1.3%
Residues in disallowed regions	9	2.9%
	----	------
Number of non-glycine and non-proline residues	306	100.0%
Number of end-residues (excl. Gly and Pro)	1	
Number of glycine residues (shown as triangles)	19	
Number of proline residues	14	

Total number of residues	340	

Based on an analysis of 118 structures of resolution of at least 2.0 Angstroms
and R-factor no greater than 20%, a good quality model would be expected
to have over 90% in the most favoured regions.

7859547_01.ps

Supplementary Figure S3: Ramachandran plot for validation of *S. typhimurium*. The residues in the most favorable region 82.4%, the allowed region 13.4% , the generously allowed region 1.3% in and the disallowed region 2.9%.

Supplementary Table S1: Details of secondary structure elements of *S. typhimurium*

Secondary Structure	SOPMA (%)
Alpha helix	57.94
3_{10} helix	0.00
Pi helix	0.00
Beta bridge	0.00
Extended strand	8.24
Beta turn	3.53
Bend region	0.00
Random coil	30.29
Ambiguous states	0.00
Other states	0.00

References:

[1] Y. Zhang I-TASSER server for protein 3D structure prediction BMC Bioinforma, 9 (2008), p. 40

[2] A. Roy, A. Kucukural, Y. Zhang I-TASSER: a unified platform for automated protein structure and function prediction Nat Protoc, 5 (2010), pp. 725-738

[3] R. Apweiler, A. Bairoch, C.H. Wu Protein sequence databases Curr Opin Chem Biol, 8 (2004), pp. 76-80

[4] R.A. Laskowski, M.W. MacArthur, D.S. Moss, J.M. Thornton PROCHECK: a program to check the stereochemical quality of protein structures J Appl Cryst, 26 (1993), pp. 283-291
